# Combined *Dusp4* and p53 loss with *Dbf4* amplification drives tumorigenesis via cell cycle restriction and replication stress escape in breast cancer

**DOI:** 10.1186/s13058-022-01542-y

**Published:** 2022-07-18

**Authors:** Ann Hanna, Mellissa J. Nixon, M. Valeria Estrada, Violeta Sanchez, Quanhu Sheng, Susan R. Opalenik, Abigail L. Toren, Joshua Bauer, Phillip Owens, Frank M. Mason, Rebecca S. Cook, Melinda E. Sanders, Carlos L. Arteaga, Justin M. Balko

**Affiliations:** 1 Departments of Medicine, Vanderbilt-Ingram Cancer Center, Vanderbilt University Medical Center, 2200 Pierce Ave, 777 PRB, Nashville, TN 37232 USA; 2 Department of Pathology, Microbiology & Immunology, Vanderbilt-Ingram Cancer Center, Vanderbilt University Medical Center, 2200 Pierce Ave, 777 PRB, Nashville, TN 37232 USA; 3 Breast Cancer Research Program, Vanderbilt-Ingram Cancer Center, Vanderbilt University Medical Center, 2200 Pierce Ave, 777 PRB, Nashville, TN 37232 USA; 4 Vanderbilt-Ingram Cancer Center, Vanderbilt University Medical Center, Nashville, TN 37232 USA; 5 Department of Early Discovery Oncology, Merck & Co., Boston, MA USA; 6 Department of Biostatistics, Vanderbilt University Medical Center, Nashville, TN USA; 7 Vanderbilt Institute of Chemical Biology, Nashville, TN USA; 8 Department of Pathology, University of Colorado Anschutz Medical Campus, Aurora, CO USA; 9 Biomedical Engineering, Vanderbilt University School of Engineering, Nashville, TN USA; 10 Simmons Comprehensive Cancer Center, University of Texas Southwester, Dallas, TX USA

**Keywords:** Breast cancer, Oncogenesis, *Dusp4*, Replication stress, p53, *Dbf4*

## Abstract

**Aim:**

Deregulated signaling pathways are a hallmark feature of oncogenesis and driver of tumor progression. Dual specificity protein phosphatase 4 (DUSP4) is a critical negative regulator of the mitogen-activated protein kinase (MAPK) pathway and is often deleted or epigenetically silenced in tumors. DUSP4 alterations lead to hyperactivation of MAPK signaling in many cancers, including breast cancer, which often harbor mutations in cell cycle checkpoint genes, particularly in TP53.

**Methods:**

Using a genetically engineered mouse model, we generated mammary-specific *Dusp4*-deleted primary epithelial cells to investigate the necessary conditions in which DUSP4 loss may drive breast cancer oncogenesis.

**Results:**

We found that *Dusp4* loss alone is insufficient in mediating tumorigenesis, but alternatively converges with loss in Trp53 and MYC amplification to induce tumorigenesis primarily through chromosome 5 amplification, which specifically upregulates *Dbf4*, a cell cycle gene that promotes cellular replication by mediating cell cycle checkpoint escape.

**Conclusions:**

This study identifies a novel mechanism for breast tumorigenesis implicating *Dusp4* loss and p53 mutations in cellular acquisition of *Dbf4* upregulation as a driver of cellular replication and cell cycle checkpoint escape.

**Supplementary Information:**

The online version contains supplementary material available at 10.1186/s13058-022-01542-y.

## Introduction

*Dual-specificity phosphatase-4* (*DUSP4*) is a tumor suppressor gene that is frequently downregulated in human cancer through both epigenetic and genomic mechanisms [1–8]. The DUSPs represent a large family of phosphatases [9] with varying tissue expression, cellular localization and serine/threonine substrates. DUSP4 exhibits specificity for JNK and ERK kinases. Although JNK and ERK dephosphorylation by multiple DUSP family members has been reported, DUSP4 has a non-redundant function in suppressing these pathways [10].

Somatic and/or epigenetic DUSP4 loss of function (LOF) has been attributed to oncogenic function in EGFR-mutant non-small cell lung cancer [5, 11], glioma [7], diffuse large B cell lymphoma [8] and breast cancer [1–4, 6]. DUSP4 is an immediate early response gene to growth factor stimulation, downstream of the EGR1 transcription factor [12], and is robustly induced following Ras/MEK/ERK activation. As such, it is thought that DUSP4 functions as a negative feedback suppressor to regulate the duration and amplitude of the Ras/MEK/ERK pathway. DUSP4 loss can thus enable unrestricted and potent MAPK signaling, resulting in increased cellular growth through cellular stress, changes in energy metabolism [12], and loss of cell cycle checkpoint control [13]. Interestingly, replication stress in Brca2-null cells activates p53 and the expression of its target genes, including senescence-inducing Ink4/Arf. This pathway modifies p53 targets, leading to enhanced p53-mediated expression of DUSP4, resulting in cellular senescence. DUSP4 blockade has been reported to bypass replication stress-induced senescence in the context of sustained MAPK signaling [14].

We and others have previously identified DUSP4 as a critical regulator of Ras/MAPK activity and cancer stem cell-like functionality in breast cancer, most notably in basal-like breast cancers (BLBC) [2–4, 13], a molecular breast cancer subtype correlating with clinically defined triple negative breast cancers (TNBCs). BLBCs are nearly always accompanied by inactivating mutations in *TP53*, encoding p53 [15]. Given the previously reported role of DUSP4 as a p53 target, a potential cell cycle checkpoint, and in mediating senescence downstream of replication stress, we hypothesized that DUSP4 may be a critical oncogenic driver in breast cancer. However, targeted deletion of *Dusp4* in mice as a single lesion is not known to generate spontaneous tumors of any kind, and these mice have mainly been used to study immunologic responses to inflammatory stimuli, such as infection [16] and cardiomyopathy, where combined deletion of both DUSP1 and DUSP4 is required for a cardiac phenotype [17].

In this study, we tested whether *Dusp4* loss, together with other oncogenic events, could promote mammary tumorigenesis. We find that together with loss-of-function mutations in p53 and, to some degree, potentiated by cMyc overexpression, *Dusp4* deletion aids in cell cycle checkpoint escape while simultaneously potentiating hallmarks of replicative stress, including multinucleation and Chk1 phosphorylation. Transformation was accompanied by focal amplification of the centromeric region of chromosome 5q, which includes cell cycle genes such as *Cdk14* and *Cdk6*, as well as *Dbf4* which controls origin firing and replication fork progression. We hypothesize that this amplification occurred due to chromosome mis-segregation during *Dusp4* loss-induced replication stress and is selected for to re-establish replication maintenance and complete cell cycle control loss through specifically *Dbf4* amplification. Finally, in both murine mammary cancer models and human breast cancers, we confirm associations of *DBF4* overexpression with p53 and *DUSP4* loss, suggesting co-dependency of these events in certain avenues to basal-like breast cancer carcinogenesis.

## Methods

### Genetically engineered mouse models

A targeting vector based on the *C57/BL6* mouse *Dusp4* genomic sequence was generated by genOway (Lyon, France), which included *loxP* sites flanking exons 3–4, the exons encoding the functional phosphatase domain and the stop codon, as well as a FRT-flanked neomycin cassette in the 3' homology arm for embryonic stem cell section. The targeting vector was transfected into *C57BL/6* embryonic stem cells followed by clonal propagation of neomycin-resistant cells. Embryonic stem cells were injected into *C57/BL6* blastocysts, which were then transplanted into *C57/BL6* pseudopregnant females. Agouti offspring were mated to FLP recombinase transgenic mice, and resulting offspring were assessed for genomic recombination at the *Dusp4* allele and neomycin cassette excision by southern analysis. Resulting mice, referred to herein as Dusp4^fl/wt^ mice, were expanded on a *C57BL/6* background and intercrossed to generate Dusp4^FLOX^ mice. No overt phenotype in male or female Dusp^FLOX^ mice was observed, including fecundity, weight, and behavior under conventional housing.

The floxed *Dusp4* allele was backcrossed for > 15 generations from *C57BL/6* to the *FVB/n* background. No overt developmental phenotype in the mammary gland was observed in *FVB/n* mice crossed to MMTV-Cre mice (Jackson Labs, data not shown), which was performed prior to crossing with the MMTV-neu-ires-cre (MMTV-NIC, Jackson Labs) *FVB/n* model. *FVB/n* Dusp4^WT/WT^ x MMTV-NIC, Dusp4^FL/WT^ x MMTV-NIC, and Dusp4^FLOX^ x MMTV-NIC female mice were monitored 3 × weekly.

For tumor growth analysis, tumors were measured twice weekly with calipers and volume was calculated in mm^3^ using the formula (length x width x width/2). Measurements reflected the combined size of all palpable tumors across mammary glands (burden). Mice were humanely euthanized when the tumor burden reached 2 cm^3^.

### Generation of derivative cell lines and cell culture

Primary mammary epithelial cells from a 6-week-old Dusp4^FLOX^ C57/BL6 mouse were isolated by enzymatic and mechanical dissociation and cultured in Cnt-Prime progenitor cell-targeted media for > 2 months according to the manufacturer’s recommendations (CELLnTEC). A population of progenitor-like epithelial cells was generated with a stable phenotype beyond 30 passages. These cells were first transiently infected with Ad-GFP or Ad-Cre-mCherry (Vector Labs) at an MOI of 100 and sorted by flow cytometry, generating Dusp4^FLOX^ and Dusp4^NULL^ cell lines. After loss of fluorescence (at least 14 days), both cell lines were subsequently infected with retrovirus from pQCIG [18] expressing sgRNA for exon 5 or 7 of *Trp53* (Trp53^Δex5/ex7^) or a control sgRNA (Trp53^WT^), sorted for GFP expression, and selected by 1uM nutlin 3a treatment for 1 week (with the exception of the control-transduced cells) to enrich functionally p53-deficient cells as previously described [18]. Each derivative cell line was then transduced with lentivirus (pLX302) containing expression cassettes for *LACZ* (MYC^WT^) or human *cMYC* (MYC^AMP^). All cell lines were maintained in Cnt-Prime media for all experiments, or until injected into the #5 mammary fat pad. Initial tumor implantation studies using progenitor cell lines were performed within 10 passages of cell line generation (i.e., the final genetic manipulation).

The DPM (Dusp4^NULL^ Trp53^Δex7^ MYC^AMP^) cell line was established by enzymatic and mechanical dissociation of a primary tumor grown in a nu/nu mouse and cultured in serum-free DMEM/F12 media containing hydrocortisone, EGF, and insulin for 1 month to deplete fibroblasts, before transitioning to DMEM + 10% FBS for routine culture. DPM cells were further transduced with pGIPZ lentivirus shRNA targeted at murine *Dbf4* (shDbf4) (Clone ID: V2LMM_219955; sequence: TGCTGTTGACAGTGAGCGCCCGAGTGCTGAATTGGATAAATAGTGAAGCCACAGATGTATTTATCCAATTCAGCACTCGGTTGCCTACTGCCTCGGA) or non-targeted control (shNTC) and selected for bright GFP expression by fluorescence-assisted cell sorting. Individual single-cell-derived clones were expanded and evaluated for knockdown by qRT-PCR for *Dbf4*. Four clones with *Dbf4* mRNA < 25% that of NTC were pooled for subsequent studies. Human DUSP4 and phosphatase-dead DUSP4 generated by site-directed mutagenesis (DUSP4-PD; C279S [19]) were generated by cloning the human DUSP4 gene coding sequence from MDA-MB-231 cells, inserted into pENTR221 by ligation and recombined into the pLX304 lentiviral vector by Gateway cloning.

### Orthotopic animal experiments

For orthotopic models, cell lines (1 × 10^6^ cells) were injected in 100uL Matrigel into the #5 mammary fat pad of athymic *nu/nu* female mice or 6-week old *C57/BL6* female mice, where indicated. Tumor formation and growth was followed for up to 60 days. Tumors were measured 2 times weekly with calipers, and volume was calculated in mm^3^ using the formula (length x width x width/2). Mice were humanely euthanized when the tumor burden reached 2 cm^3^, or at 60 days, if tumors had not yet reached this endpoint or if no tumor formed. Initial tumor implantation studies were performed within 10 passages of cell line generation (i.e., the final genetic manipulation).

### Soft agar assays

Soft agar assays were carried out in 6-well dishes using 5 × 10^4^ cells. A single-cell suspension in 0.4% agarose in 1 × media was layered on the top of a bottom layer of 0.8% agarose in 1 × media. Fresh 1 × media was applied to cells every 3 to 4 days to protect against dehydration. Colonies were examined after 2 to 3 weeks on a GelCount Scanner (Oxford Optronix).

### Western blotting

Western blotting was performed as previously described [20]. Briefly, tumor fragments or cells were homogenized in 1X RIPA buffer lacking SDS detergent (50 mM Tris pH 7.4, 150 mM NaCl, 1.0% NP-40, 0.5% deoxycholic acid, 1 mM EDTA, 1 mM EGTA, 5 mM sodium pyrophosphate, 50 mM NaF, 10 mM b-glycerophosphate) with added phosphatase inhibitors (PhosSTOP, Roche) and protease inhibitors (cOmplete, Roche) using a Qiagen TissueLyser. Lysates were adjusted to 0.1% SDS, followed by 30-min incubation on ice. Lysates were centrifuged at 13,000xg for 15 min at 4 °C. Protein concentrations of the lysates were determined by BCA assay (Thermo). Samples were separated by 10% SDS-PAGE and transferred to nitrocellulose membranes. Membranes were blocked with 5% non-fat dry milk or 5% BSA in tris-buffered saline (TBS) with 0.1% Tween-20 for 1 h at room temperature and then incubated overnight at 4 °C with the appropriate primary antibody in blocking buffer as indicated. Following incubation with appropriate horseradish peroxidase-conjugated secondary antibodies, proteins were visualized using an enhanced chemiluminescence detection system (Thermo). This study was performed using the following antibodies: AKT (2920; Cell Signaling Technology), Calnexin (SC-11397; Santa Cruz), cMYC/nMYC (13,987; Cell Signaling Technology), HDAC1 (5356; Cell Signaling Technology), cJUN (9165; Cell Signaling Technology), DUSP4 (5149; Cell Signaling Technology), ETS-1 (SC-350, Santa Cruz), p21 (6246; Cell Signaling Technology), Phospho-AKT(Ser473) (4060; Cell Signaling Technology), Phospho-CHK1 (Ser345) (2348S; Cell Signaling Technology), Phospho-cJUN (Ser63) (2361; Cell Signaling Technology), Phospho-cJUN (Ser73) (3270; Cell Signaling Technology), Phospho-ERK1/2 (9120; Cell Signaling Technology), Phospho-SAPK/JNK (9255; Cell Signaling Technology), and RPA32 (52448S; Cell Signaling Technology).

### Multiplexed immunofluorescence

Paraffin tissues were embedded and sectioned at 5 μM and dewaxed in xylene and rehydrated in alcohol with citrate antigen retrieval. Standard Mayer’s hematoxylin and eosin (H&E) was performed. The following antibodies were used: Krt8/18 (Fitzgerald 20R-CP004 1:500) and Krt5 (BioLegend 905,501 1:500). Paraffin-derived sections were counterstained with hematoxylin (Vector Labs) and mounted with Cytoseal. Immunofluorescence staining was performed with primary and secondary antibodies diluted in 12% Fraction-V BSA (Pierce) and slides mounted in SlowFade mounting medium containing DAPI (Invitrogen). All fluorescent secondary antibodies (Krt5: Goat Anti-Rabbit A488 A-11034 (K8/18: Goat Anti-Guinea-Pig A594 A-11076, Thermo Fisher) were highly cross-adsorbed and used at a dilution of 1:200 for 20 min. Nuclei were stained with SlowFade Gold with DAPI (S36939 Thermo Fisher).

### Quantitative real-time PCR

RNA was harvested from cells or tumor homogenates using the Maxwell 16 automated workstation (Promega) and LEV simply RNA Tissue kit (Promega). RNA was then analyzed for concentration by a NanoDrop 2000 (Thermo Fisher) prior to cDNA synthesis using the SensiFAST cDNA synthesis kit (Bioline) with 1 µg of RNA per sample. cDNA and SSO advanced SYBR green universal supermix (BioRad) were then combined with target-specific primers on a CFX96 Touch Real-Time PCR Detection System (BioRad). Oligo sequences for qRT-PCR consisted of: *Akap9* forward 5’-TTACCATTGCAGAATAGGTACCCG-3’ reverse 5’-AACGGATTATCTCCTCATGCC-3’; *Cdk6* forward 5’-TGGTCAGGTTGTTTGATGTGTGC-3’ reverse 5’-AGTCCAGACCTCGGAGAAGC-3’; *Cdk14* forward 5’-TTGTCCGAGAGTTTCAGCCG-3’ reverse 5’-TTGTGACACATATCTCATCAAAGGT-3’; *Dbf4* forward 5’-ACGAAGATCTCGAAACTCACC-3’ reverse 5’-AAGAAAGGGACCCGACACTG-3’; *Dusp4* forward 5’CATCGAGTACATCGACGCAG-3’ reverse 5’-ATGAAGCTGAAGTTGGGCGA-3’; *Fzd1* forward 5’-GAGGTGCACCAGTTCTACCC-3’ reverse 5’-TCACACTTGAGCGTGTCTGG-3’; *Gapdh* forward 5’-AGGTCGGTGTGAACGGATTTG-3’ reverse 5’-TGTAGACCATGTAGTTGAGGTCA-3’.

### Clonogenic growth assay

PMECs transduced with CRISPR/Cas9 constructs targeting Trp53 (exon 7 or exon 5) or scramble control (Parent) were plated at a density of 1,000 cells per well in a 6-well plate and treated with 10uM nutlin-3a or DMSO for 7 days. Cells were fixed and stained with 0.5% crystal violet for 20 min at room temperature.

### High-content DNA analysis

Fixed, DAPI-stained cells were imaged using a 10 × Nikon Plan Fluor objective and DAPI filter on the ImageXpress Micro Widefield High Content Screening System (Molecular Devices) in the Vanderbilt High-throughput Screening (HTS) core facility. Images were analyzed using the Micronuclei application module within the MetaXpress software. This analysis module identifies individual Hoechst-stained nuclei based on the size, intensity, and distance from adjacent cells. The nuclei from the total number of cells in the well are classified as mononucleated, binucleated, multinucleated, or mitotic. Micronuclei are identified based on the size, intensity, and distance from the main nucleus. A small nuclear mass that is contiguous or attached to a main nucleus was not identified as a micronucleus. Nine sites were captured per well, which contained a minimum of 1000 cells per condition. Data are expressed as the mean % multinucleated cells from 3 replicate cultures ± standard deviation. Statistical significance of the % multinucleated cells in the treated cultures at each dose level compared with the control (vehicle treated) cultures was determined using a *t* test. The micronuclei application module automatically excludes mitotic and apoptotic nuclei (proprietary algorithm). Calculated values were exported from MetaXpress, and data were plotted using GraphPad Prism software.

### Whole-genome sequencing

DNA isolated from murine cell lines was sequenced to 10X coverage on an Illumina HiSeq. FastQC was performed on raw data, Cutadapt for adapter trimming, BWA v0.7.12 for genome mapping, and base recalibration based on GATK3 (v3.5.0). cnMops was used to identify copy number variation. DNA was harvested from progenitor cell lines within 10 passages of cell line generation (i.e., the final genetic manipulation).

### Cell cycle analysis

EdU incorporation was measured using Click-iT EdU Alexa Fluor 488 Flow Cytometry Assay Kit (Thermo Fisher) according to manufacturers’ protocol. Briefly, cells were labeled with 10 uM Click-iT EdU for 2 h in normal growth media, harvested, fixed for 15 min at room temperature, and permeabilized. Cells were stained with EdU Click-iT reaction detection cocktail for 30 min at room temperature, covalently catalyzing the Alexa Fluor 488-conjugated picolyl azide and ethynyl moiety of EdU. Finally, cells were stained with DAPI for 15 min for DNA content quantification. Flow cytometry was performed on samples using a Nxt Attune Analyzer (Thermo Fisher), and files were analyzed with FlowJo software.

### Fluorescence in situ hybridization

A custom FISH probe was generated from bacterial artificial chromosome (BAC) Clone Library RPCI-23 (clone name RP23-22E16) labeled with Green 5-Fluorescein dUTP covering the predicted amplified region of murine (mm10) chromosome 5 containing the *Dbf4* gene. FISH hybridization and analysis was performed according to the manufacturer’s recommended protocol (Empire Genomics). Deparaffinization, protease treatment, and washes were performed as per standard protocols. After this pretreatment, 4-μm FFPE tissue sections were denatured in the presence of 10  μL of the probe for 6 min at 72 °C and hybridized at 37 °C overnight in StatSpin (ThermoBrite, Abbott Molecular) with the FISH probe RP23-22E16. Post-hybridization saline sodium citrate washes were performed at 72 °C and the slides were then stained with DAPI before analysis. Tumor tissue was scanned at 20X magnification (BX60 fluorescent microscope, Olympus) to identify appropriate regions for analysis. Images for cell counting were captured with a 100 × oil immersion objective using CytoVision software (Leica). At least 40 tumor cells per case were scored.

To generate metaphase spreads, cells were treated with 150 ng/mL colcemid (Gibco KaryoMAX in PBS) for 1.5 h, harvested with trypsin, and swollen in 1:1 solution of 75 mM KCl/0.9% sodium citrate for 10 min at 37 °C. Cells were pelleted, then fixed with a 3:1 ice cold solution of methanol/acetic acid, added dropwise while gently vortexing. Following fixation, cells were pelleted and resuspended in fresh fixative and dropped onto humidified slides and air-dried overnight. FISH was performed following manufacturer’s protocol (Empire Genomics), stained with DAPI, and mounted with ProLong Antifade Gold (Thermo Fisher) under #1.5 glass coverslips. Metaphases were imaged using a 100 × 1.4 NA objective (Olympus) on a DeltaVision Elite imaging system (GE Healthcare) equipped with a Cool SnapHQ2 charge-coupled device (CCD) camera (Roper). Optical sections were collected at 200-nm intervals and processed using ratio deconvolution in softWoRx (GE Healthcare). Images are from single z-slices and were prepared for publication using ImageJ (Version 2.1.0/1.53 h).

### Statistical analysis

Statistics were performed in GraphPad Prism or R (www.r-project.org). In data with two groups, two-sample t tests were utilized. For analyses with > 2 groups, significant differences were determined by ANOVA with a Tukey’s post hoc test adjustment for multiple comparisons. For all multiple comparisons, statistical significance is noted by *p < 0.05; **p < 0.01, ***p < 0.001 and ****p < 0.0001. A *p*-value of < 0.05 was considered statistically significant. Bar graphs show mean ± SEM, unless otherwise stated in the figure legend.

## Results

### *Dusp4* loss in MECs does not accelerate MMTV-neu-mediated tumorigenesis

**Fig. 1:**
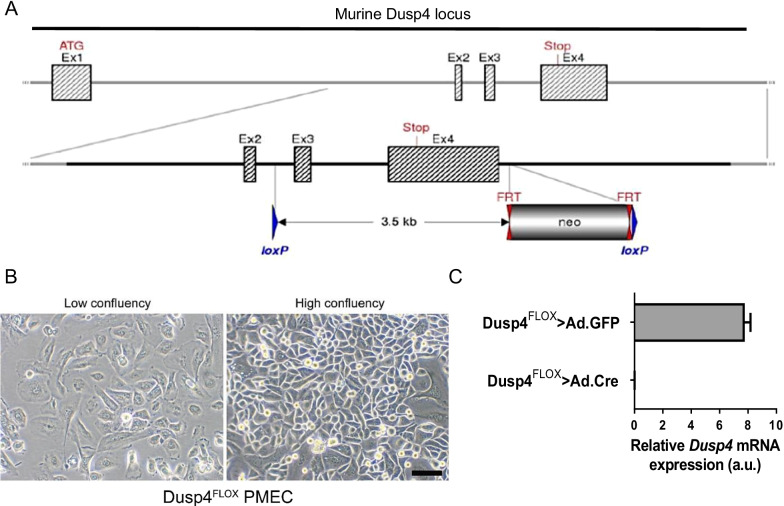
Generation of *Dusp4*-null progenitor-like primary mammary epithelial cells. **A** Schema for generation of a floxed murine *Dusp4* allele. loxP sites flanking exons 3–4 were inserted using homology-directed recombination. The FRT-flanked neo cassette in the 3’ homology arm was excised following crossing to Flp deleter mice. **B** Bright-field images of Dusp4FLOX cells at 40X (scale bar = 50 µm), low and high confluency. **C** *Dusp4* mRNA expression in Dusp4FLOX cells after transient infection with Ad.GFP or Ad.Cre

We and others have previously shown that DUSP4 is a potential tumor suppressor in basal-like or TNBC and is frequently lost or downregulated, leading to cancer stem-like phenotypes and activation of MAPK pathways [2–4, 13]. *Dusp4* has also been described as a direct target of p53 [14, 21] and a G1/S checkpoint modulator [13]. However, the role of *Dusp4* in tumor initiation has not been studied. To address this, while avoiding impact of *Dusp4* loss on immunologic function [16, 22], we generated a murine model with a floxed *Dusp4* allele (Fig. 1A). Floxed *Dusp4* mice were crossed with the MMTV-Neu-Ires-Cre (MMTV-NIC) luminal model of mammary tumorigenesis, which ensures *Dusp4* deletion in Neu + tumor cells. MMTV-NIC is a useful model to study the contribution of loss of tumor suppressor genes, such as *PTEN*, in breast cancer development [23]. Interestingly, Dusp4^FLOX^ MMTV-NIC tumors did not display shorter latency or accelerated growth rates; in fact, bi-allelic deletion modestly prolonged latency (Additional file 1: Fig S1A*; *in vitro proliferation of the cell lines depicted in Additional file 1: Fig S6A). *Dusp4* mRNA was concordantly decreased in tumors from heterozygous and homozygous animals, and biallelic deletion resulted in modestly upregulated ERK1/2 and JNK phosphorylation in tumors, the expected and known targets of *Dusp4* phosphatase activity (Additional file 1: Fig S1B–C).

We reasoned that this lack of phenotype could be due to the luminal-like nature of the MMTV-Neu model, given that most examples of DUSP4 loss occur in basal-like breast cancer [3, 4], which is substantially different in nature to that of luminal like disease [15]. As preliminary studies of *Dusp4* loss alone in the mammary gland via MMTV-Cre crosses did not produce mammary gland hyperplasia (data not shown), we concluded that alternative oncogenic events more replicative of those observed commonly in basal-like breast cancer may cooperate differently with *Dusp4* loss.

Two hallmark oncogenic events enriched in basal-like breast cancer are *Tp53* (p53) loss-of-function mutations [15] and *MYC* amplification [24]. Interestingly, in human breast cancer [15], *DUSP4* deletion events are highly co-occurring with both of these lesions (Additional file 1: Fig. S2A). Deletion of *Dusp4* in the context of *Trp53*^*−/−*^ models in a mammary tissue-specific manner would represent a technically challenging approach, and thus we asked whether the contribution of these genomic events to mammary cell transformation could instead be studied through genetic manipulation of mammary-derived progenitor-like cells from the glands of *Dusp4*^*FLOX*^ mice.

To this end, we generated a *C57/BL6 Dusp4*^*FLOX*^ cell line from mammary epithelial cells using a fully defined, animal-component-free culture medium for isolation and expansion of epithelial cells, which includes growth factors and co-factors to extend progenitor cell longevity and improve growth factor binding to membrane-bound receptors. These primary mammary epithelial cells (PMECs) demonstrated moderate growth rates, tight junctions at high confluency (Fig. 1B), and would continue in culture for > 30 passages (limit not formally reached). Using these cell lines, *Dusp4* could be excised and complete loss of *Dusp4* mRNA was observed by transient adenoviral infection with Cre (Fig. 1C). Subsequent genetic events in the cells, as detailed throughout the remainder of this report, were introduced by lenti- and retroviral transduction as shown in Additional file 1: Fig S2B.

### *Dusp4* loss cooperates with Trp53 loss of function to promote anchorage-independent growth

**Fig. 2:**
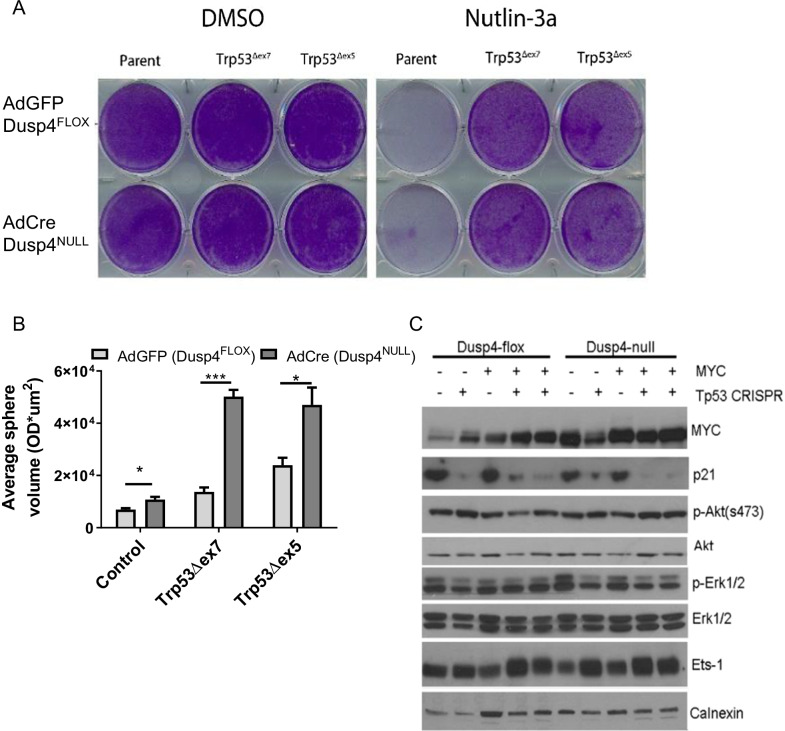
Enhanced soft agar growth with combined *Dusp4* loss and Trp53 (p53) deletion. **A** Crystal violet 2D culture assay of PMECs transduced with CRISPR/Cas9 constructs targeting Trp53 (exon 7 or exon 5) or scramble control (Parent) and selected with 10uM nutlin-3a for 1 week, or DMSO control. Trp53 CRISPR transductants demonstrate insensitivity to MDM2 inhibition, verifying loss of p53 function. **B** Soft agar colony formation assay for Dusp4FLOX or Dusp4NULL PMECs transduced with control sgRNA or Trp53 sgRNA. **C** Western blot analysis of derivative PMECs

To generate derivatives of both *Dusp4*-competent and deleted PMECs (*Dusp4* mutations shown in Additional file 1: Fig SA3A–B), we utilized a functional selection strategy with *Trp53*-targeted CRISPRs. Cell lines were transduced with either one of two sgRNA/Cas9 retroviruses targeted to exon 5 or 7 of *Trp53* (or scrambled control). Transduced cells were selected with nutlin-3a (MDM2 inhibitor, not used for scrambled control cells) for 1 week to generate polyclonal cell lines with p53 loss of function (LOF; Fig. 2A, deletion confirmed by immunoblotting Additional file 1: Fig S3D) according to previous studies [18]. Disruptive insertions/deletions in the p53 gene at the appropriate target exons were confirmed by sequencing (data summarized in Additional file 1: Fig S3C). Soft agar colony-forming studies showed a modest increase in colony size distribution with both *Dusp4* loss and p53 LOF, that appeared to be more dramatic in cell lines with both deletions, suggesting possible transformation (Fig. 2B).

We next performed subsequent transduction of enforced MYC or LACZ control into each cell line. Western blot analysis confirmed loss of p21 expression (a canonical p53 target) and overexpression of MYC in appropriate cell lines, but little other effects on MAPK or PI3K pathway signaling in whole cell lysates were observed (Fig. 2C). Interestingly, modest (and sometimes inconsistent) overexpression of MYC was observed with *Dusp4* loss, although the mechanism underlying this observation was not determined or tested in this study.

### *Dusp4* loss cooperates with Trp53 loss of function and MYC amplification to promote tumorigenesis

**Fig. 3:**
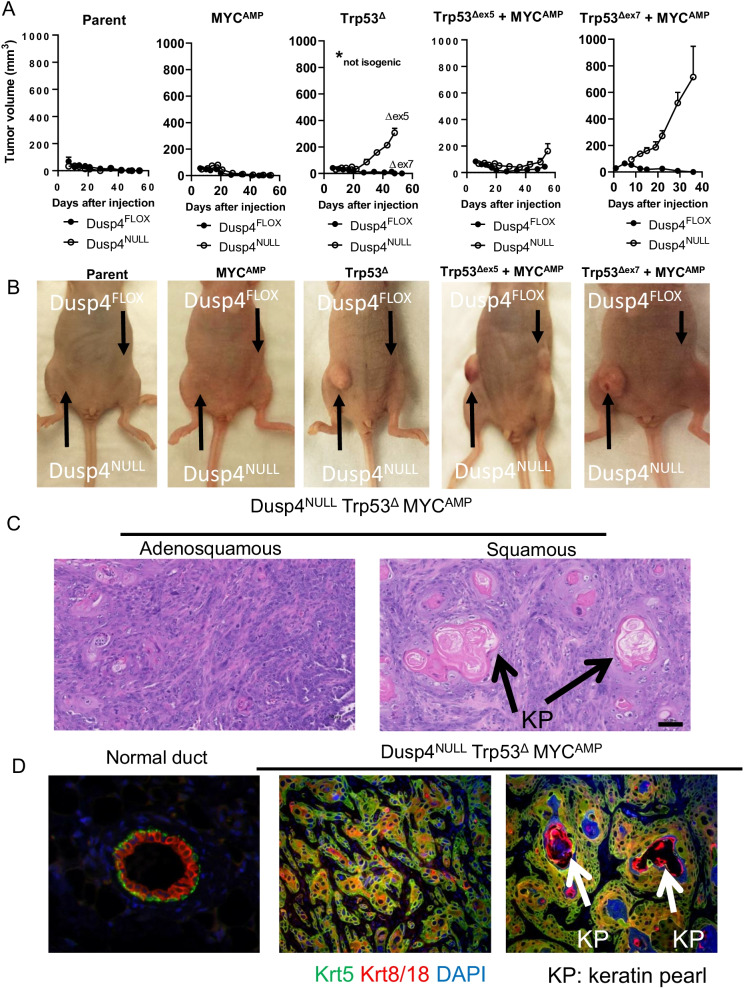
*Dusp4* loss potentiates tumorigenesis in cooperation with p53 loss and MYC overexpression. **A** Tumor formation and growth rate of orthotopically transplanted PMECs bearing *Dusp4* loss, Trp53 loss, and/or MYC amplification. *Dusp4*-competent and excised comparator cell lines in each case were transplanted into opposite mammary fat pads (*n* = 5 mice/group). Dusp4FLOX Trp53Δex5 and Dusp4NULL Trp53Δex7 derivatives were lost early in development due to contamination and thus are not isogenic with one another, but bear similar functional genomic modifications. **B** Example images of nu/nu mice bearing *Dusp4*-competent and excised comparator cell line inoculations in opposing mammary fat pads. **C** Representative images of H&E histology of tumors derived from Dusp4NULL Trp53Δ MYCAMP cells with adenosquamous or squamous differentiation. KP: keratin pearls. **D** Multiplexed immunofluorescence for basal (Krt5) and luminal (Krt8/18) keratins in tumors arising from Dusp4NULL Trp53Δ MYCAMP cells, with an associated normal duct as a control for staining. KP: keratin pearls

To more directly study the effect of *Dusp4* loss on tumorigenic features of the cell line derivatives, we orthotopically implanted each cell line derivative with its appropriate *Dusp4*-competent and *Dusp4*-deleted isogenic derivative in opposing #5 mammary fat pads of athymic nu/nu mice. Tumor formation and growth were monitored for 60 days. Strikingly, we observed robust tumor formation and cell growth with *Dusp4* loss in a p53 LOF context, regardless of *MYC* amplification (Fig. 3A–B). This observation was consistent with our in vitro soft agar growth assays (Fig. 2B). Although a direct contribution of MYC overexpression could not be determined due to a lack of completely isogenic pairs, it was of note that the most aggressive cell line also harbored MYC amplification. Only one cell line with competent *Dusp4* produced tumors (Trp53^Δex5^ + MYC^AMP^), and these tumors were substantially smaller and slower growing than their *Dusp4*-deleted counterparts. The resulting tumors demonstrated primarily squamous and adenosquamous histologies, with the presence of robust keratin pearls (KP; Fig. 3C). These tumors also expressed both basal (keratin 5) and luminal (keratin 8/18) cytokeratins in heterogeneous patterns (Fig. 3D). Tumors were dissociated, and primary tumor cells were cultured for molecular analysis of transformed cells driven by defined genetic alterations. Thus, the **D**usp4^NULL^/Tr**p**53^Δex7^/**M**YC^AMP^ (DPM) tumor cell line was used for subsequent study.

### Acute *Dusp4* loss does not directly impact tumorigenesis and is uncoupled from phosphatase activity

**Fig. 4:**
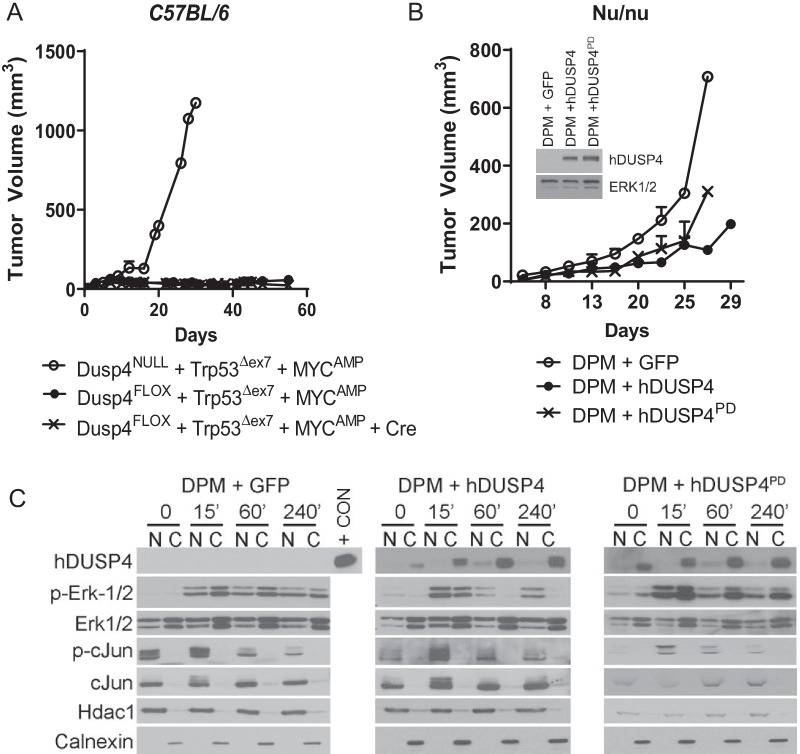
Evidence that tumor-forming potential of Dusp4NULL Trp53Δ MYCAMP cells is not due to classical *Dusp4* function. **A** Tumor growth curves of Dusp4NULL Trp53Δex7 MYCAMP cells (tumor forming) or Dusp4FLOX Trp53Δex7 MYCAMP cells (not tumor forming), with or without Cre, administered 1 week prior to injection into C57/BL6 mice. (*n* = 5 mice/group) **B** Tumor growth curves of DPM tumor cell line (derived from re-culture of dissociated Dusp4NULL Trp53Δex7 MYCAMP tumors), after transduction with GFP control, or reconstitution of human DUSP4, or phosphatase-dead DUSP4 (hDUSP4PD) (*n* = 5 mice/group). Inset demonstrates expression of DUSP4 by a human-specific antibody. **C** Nuclear and cytoplasmic fractions of DPM GFP, hDUSP4, and hDUSP4PD cell lines, serum starved and stimulated with FBS over a time course. + CON is human MDA-MB 231 cell lysate

Since we observed a robust effect of *Dusp4* loss on tumorigenic potential, coupled with modest changes in Ras/MAPK pathway activity, we sought to determine whether direct canonical *Dusp4*-mediated phosphatase activity and Ras/MAPK signaling was indeed the primary factor in tumorigenesis. To test this canonical hypothesis, we utilized *Dusp4* competent Trp53^Δex7^ + MYC^AMP^ cells, which did not form tumors, and acutely deleted *Dusp4* with Ad-Cre one week prior to injection into *C57/BL6* hosts. Interestingly, these cells failed to form tumors, in contrast to the cell line with de novo deficient *Dusp4* (Fig. 4A). Moreover, lentiviral reconstitution of *DUSP4* (human sequence) or phosphatase-dead *DUSP4* (DUSP4^PD^) [19] into *Dusp4*-deficient DPM tumor cells did not suppress tumor formation in *nu/nu* mice, though growth rate was modestly impacted (Fig. 4B). Re-expression of DUSP4, but not the PD-DUSP4, suppressed cytoplasmic ERK1/2 phosphorylation after serum stimulation demonstrating the predicted functionality of the re-expressed constructs (Fig. 4C). Thus, our data argue against a direct role of oncogenic cell signaling in driving tumorigenesis with *Dusp4* loss.

### Tumorigenic *Dusp4* and Trp53 loss (± MYC amplification) cell lines acquire a centromeric amplicon on chromosome 5

**Fig. 5:**
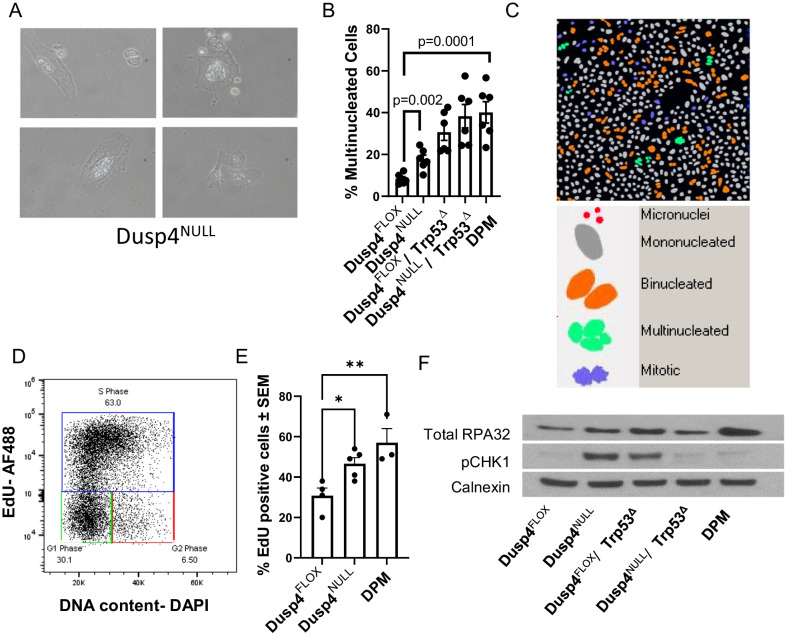
Combined *Dusp4* and p53 loss induces multinucleation and eventual escape from replication stress. **A** Representative images of polyploidy/multinucleation in *Dusp4* NULL cells. **B** Quantification of nuclear content in primary mammary epithelial-derived cell lines with various genetic alterations. **C** Representative images of Dusp4NULL cells stained with DAPI to visualize nuclear DNA content. **D** Cells were labeled with EdU and counterstained with DAPI and analyzed by flow cytometry to analyze DNA synthesis. Representative flow cytometry gating is shown. **E** S phase analysis by EdU *Tukey’s post hoc *p* < 0.05** *p* < 0.001. **F** G2 phase analysis demonstrating increased G2 phase in Dusp4NULL p53Δ and DPM cells. **G** Whole-cell lysates were immunoblotted for replication stress marker pCHK1

Given the observation that abrupt Cre-mediated *Dusp4* excision from the non-tumorigenic Dusp4^FLOX^ Trp53^Δ^ MYC^AMP^ was not sufficient to induce tumorigenesis (Fig. 4A), we postulated that *Dusp4* loss may alternatively induce cellular changes that create a selective force for the emergence of tumorigenic clones (for example, genomic instability). Supporting this possibility, multinucleation in cells was observed with *Dusp4* loss (Fig. 5A) and quantification of this across Dusp4^NULL^ and Trp53^Δ^ cell lines demonstrated enhanced multinucleation with either or both events (Fig. 5B–C). *Trp53* loss is known to induce tetraploidy, which is consistent with this observation. We hypothesized that this multinucleation could be due to replication stress, eventually leading to chromosome mis-segregation and genomic copy number variations that are selected for with in vitro propagation. Consistent with this hypothesis, loss of *Dusp4* alone increased EdU incorporation (S phase) and further increased in DPM cells (Fig. 5D–E). Supporting a possibility of enhanced DNA synthesis and replication stress, phosphorylation of Chk2 was increased in Dusp4^NULL^ cells and Trp53^Δ^ cells, indicating replication stress that was relieved in cells carrying both alterations (Fig. 5F).

**Fig. 6:**
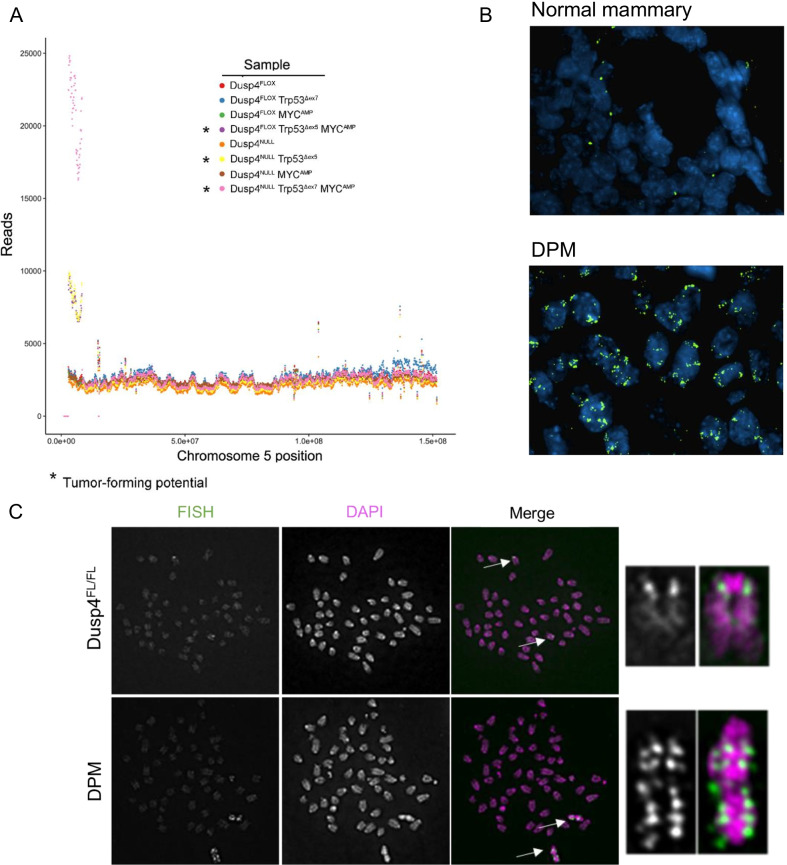
Tumorigenic capacity of Dusp4NULL Trp53Δ MYCAMP cells is associated with chromosome 5 amplification. **A** Whole-genome sequencing of PMEC-derived cell lines with *Dusp4* loss, Trp53 loss, and/or MYC amplification. Dusp4NULL Trp53Δex7 MYCAMP cells (tumor forming) or Dusp4FLOX Trp53Δex7 MYCAMP cells (not tumor forming). **B** FISH for chromosome 5 centromeric amplicon in DPM tumors compared to normal murine mammary gland tissue. **C** Representative images of FISH in metaphase spreads from DPM and Dusp4FLOX cells. In the right panel is a magnification of chromosome 5

Next, we performed low-pass (10X coverage) whole-genome sequencing (WGS) on cell line derivatives. Aside from sporadic low-level copy gains, only one focal amplification was observed in some cell lines, which encompassed the p arm (telo/acrocentric), centromere, and centromere–proximal region of murine chromosome 5 (Fig. 6A and Additional file 1: Figure S4). This amplified region, which was present only in cell line derivatives that formed tumors in nu/nu mice (Fig. 6A), included several characterized genes (*Fzd1, Akap9*), as well as cell-cycle-associated genes (*Dbf4, Cdk6, Cdk14*) (Additional file 1: Fig S5A**)**. qPCR revealed that each of these genes was highly overexpressed in amplified/tumorigenic cell lines, to differing degrees (Additional file 1: Fig S5B), including the one *Dusp4*-competent cell line (Dusp4^FLOX^ Trp53^Δex5^ MYC^AMP^) that formed small (< 300mm^3^) tumors. Amplification of this region was further confirmed by FISH (Fig. 6B) in DPM cell forming tumors compared to normal mammary tissue. Using metaphase spreads and FISH, we found that this amplification was confined to chromosome 5 with what appeared to be tandem duplications of the region throughout the same chromosome, supporting an idea of chromosome mis-segregation as a repeated event (Fig. 6C). It is unclear if this was an early or late event in the generation of the cell line models but occurred within 10 passages of the final genetic manipulation of the cell lines, as was the point of DNA harvest for NGS. Interestingly, a similar spontaneous amplification was previously reported to be observed in a C57/BL6 chimeric murine model of colorectal cancer driven by mutant p53 and beta-Catenin [25]. The presence of this amplification, which was associated with tumor propagation and metastasis, co-occurred with a spontaneous *Kras* mutation [25], thus including two of the pathway-directed alterations in our own studies (Ras/MAPK and p53).

### *Dbf4* is a driver of cell cycle checkpoint escape and tumorigenesis

**Fig. 7:**
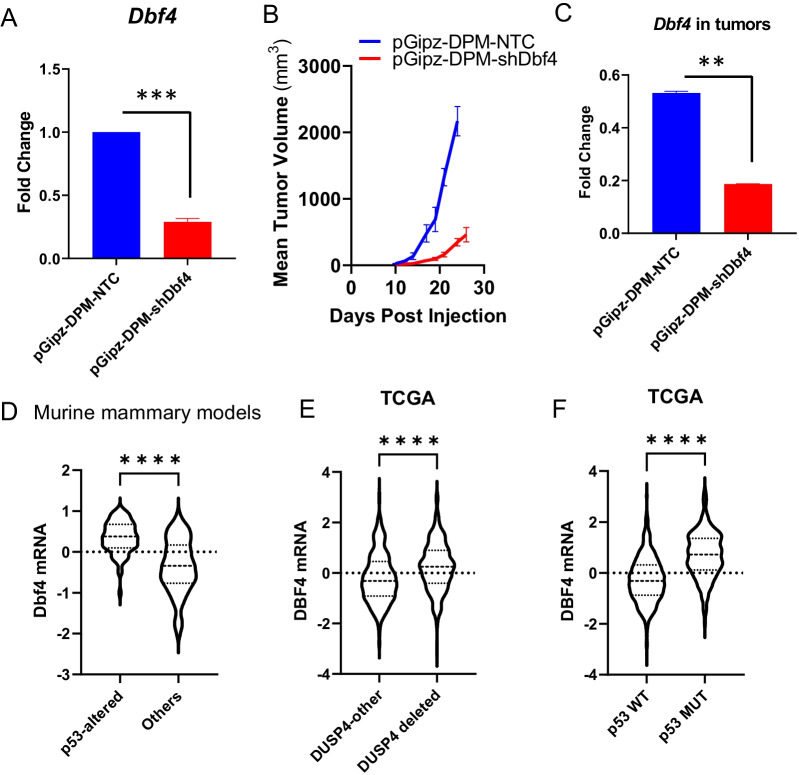
DBF4 overexpression contributes to tumor progression and occurs preferentially in p53-altered tumors and DUSP4-deleted tumors. **A** qRT-PCR analysis for *Dbf4* expression in DPM cells transduced with short-hairpin *Dbf4* or nontarget control plasmid in athymic nu/nu mice **B** Tumor growth rate of orthotopically transplanted DPM cells (*n* = 5 mice/group) transduced with short-hairpin *Dbf4* or nontarget control plasmid in athymic nu/nu mice. **C** *Dbf4* mRNA expression in DPM (control and shDbf4) tumors excised from athymic nude mice demonstrating continued *Dbf4* suppression. Interrogating murine tumor line and human TCGA databases correlates *Dbf4* mRNA overexpression with p53 mutations in (**D**) murine mammary tumors and *Dusp4* loss (**E**) and p53 mutations (**F**) in humans

Next, we hypothesized that *Dusp4*-mediated G1/S checkpoint loss may lead to changes in genomic stability leading to emergence of tumorigenic clones. Given the upregulated transcriptional signature of genes located in the amplified region of chromosome 5, we tested the potential impact of cell cycle checkpoint Cdk6, which is present within the amplified region of chromosome 5, as a potential driver of the proliferative advantage in the *Dusp4*-deleted cell lines. We found that treatment with Palbociclib, a selective CDK4/6 inhibitor, did not increase the proliferative capacity of *Dusp4*-null cells (Additional file 1: Fig S6B). Consequently, we focused on *Dbf4* as a potential driver of the transformative “switch” in DPM cells. DBF4 is a critical cell cycle regulator that forms a complex with Cdc7 (Dbf4-dependent kinase, or DDK) to initiate DNA replication and may be a “chokepoint” or bottleneck in cells rapidly progressing through G1/S by rescuing stalled replication forks [26]. Therefore, we silenced *Dbf4* expression in DPM tumor-forming cells using short-hairpin RNA (Fig. 7A) and inoculated them orthotopically in athymic nu/nu mice. Compared to nontarget control DPM cells, *Dbf4* loss significantly suppressed the tumor forming capacity by delaying tumor onset and reducing tumor growth rate (Fig. 7B). Sustained *Dbf4* knockdown in DPM-shDbf4 tumors was validated by qRT-PCR for *Dbf4* expression in excised tumors (Fig. 7C**)**.

Consistent with our observations in this study, we found that p53-altered mammary tumors demonstrated higher *Dbf4* mRNA expression in a series of 201 microarray samples from diverse genetically engineered mouse models (GEMM) of breast cancer [27] (Fig. 7D). *Dusp4* mRNA could not be measured in this dataset as it was a poor-quality probe which was filtered in QC steps. However, in The Cancer Genome Atlas (TCGA) human breast cancer dataset, both tumors with DUSP4 deletions and those with p53 loss showed elevated transcript levels of *Dbf4* (Fig. 7E–F**),** supporting cooperation among *Dbf4*, p53, and *Dusp4* in promoting breast cancer oncogenesis.

## Discussion

Oncogenic signaling is critical for tumorigenesis of nascently transformed cells. A plethora of studies demonstrate the role of MAPK/ERK signaling in promoting tumorigenic functions such as proliferation, epithelial–mesenchymal transition, and angiogenesis [28, 29]. While canonical mutations in Ras/MAPK genes (for example *KRAS G12V* and *BRAF V600E*) are rare in breast cancer, Ras/MAPK is often overactivated in response to pathway deregulation [3, 4]. Indeed, several studies from our laboratory and others implicate *DUSP4* loss in prolonged MAPK/ERK activation, particularly in highly aggressive subtypes of breast cancer, like basal-like breast cancers [3, 4]. In this study, we explored the tumorigenic potential of primary mammary epithelial cells harboring different genetic alternations and found that *Dusp4* loss converges with p53 mutation and, to a lesser extent, cMyc overexpression, to promote tumorigenesis, which was associated with chromosome 5 amplification. The amplified region of chromosome 5 contains several critical cell cycle regulators, including *Dbf4*. Importantly, this amplicon was also generated in one cell line with intact *Dusp4* which has acquired a hyper-mutated phenotype by whole exome sequencing (> 2000 SNVs; data not shown), leading to the hypothesis that while *Dusp4* loss may have facilitated the generation or selection of the chromosome 5 amplicon, it was not required, and other mechanisms could lead to the same effect.

The Cdc7-Dbf4 complex phosphorylates the minichromosome maintenance (MCM) protein complex leading to the progression of the cell cycle through S phase to induce mitotic DNA replication in mammalian cells [30]. Studies have shown that Cdc7-Dbf4 (Dbf4-dependent kinase; DDK) overrides replication stress in cancer cells to promote replication [31], which can be reversed using DDK inhibitors to promote fork stalling and cell cycle arrest [26]. To date, only one study investigated the overexpression of Cdc7-Dbf4 in many in vitro tumor lines, linking this overexpression with p53 mutations [32], albeit specific overexpression of DBF4 in primary human breast cancer was not confirmed. We found that cells with *Dusp4* and p53 loss acquire *Dbf4* amplification presumably through selective pressure, possibly to bypass fork stalling and advance to G2/M phase of the cell cycle, thus explaining their tumorigenic potential versus cells with *Dusp4* loss alone.

It is important to note that our approach has several caveats, particularly in respect to the well-studied p53 pathway. Firstly, we used a CRISPR/Cas9 p53-targeted approach that generated indels in the p53 gene, most likely generating loss of function (LOF) of p53 via truncated protein products. Both LOF and gain-of-function (GOF) mutations constitute a considerable proportion of TP53 alterations in human breast cancer, though GOF appears to be moderately more prevalent. Thus, our approach was not designed to dissect differences between these types of p53 alterations. However, recent studies have found that most of the GOF phenotypes thought to be directly mutant p53-mediated are, in fact, related primarily to the aneuploidy and genomic entropy caused by p53 LOF [33]. Secondly, we used pharmacologic selection with nutlin-3a (7 days) to select p53 LOF populations within our cell line models. By nature, this approach did not permit us to treat non-p53-targeted cell lines with nutlin-3a. Other p53-independent effects could also occur with nutlin-3a as well; however, our resulting selected subclonal populations had confirmed high allelic frequency p53 alterations, suggesting that CRISPR-mediated LOF was the primary mechanism of nutlin-3a resistance. Despite these caveats, to our knowledge, this is the first report linking *Dusp4* loss and p53 with *Dbf4* overexpression in murine mammary tumors as a novel mechanism for oncogenesis in breast cancer.

## Conclusions

The regulation of oncogenic signaling pathways is critical for preventing tumorigenesis. In this work, we created murine models with mammary-specific *Dusp4* deletions to study the mechanisms of DUSP4-mediated transformation of mammary epithelial cells that drive breast oncogenesis. We discovered that *Dusp4* deletions only drive oncogenesis when accompanied by other multigenic events, such as Trp53 loss and MYC amplification. The combined mutations amplify the centromeric region of chromosome 5q, which encodes *Dbf4*, a critical cell cycle gene that promotes cellular replication, to permit the cell cycle checkpoint escape of transformed cells.

## Supplementary Information

**Additional file 1.** **Fig** **1**: Dusp4 hemi- or homozygous deletion does not accelerate MMTV-neu-mediated tumorigenesis or growth. A) Kaplan–Meier survival of FVB/n NIC mice with Dusp4WT (n=8), Dusp4FLOX/WT (n=11), or Dusp4FLOX (n=10) alleles. Time from birth to tumor endpoint (2cm3 total tumor burden), time from birth to first palpable tumor, and time from palpable tumor to tumor endpoint are shown. B) Dusp4 mRNA expression measured from bulk tumor RNA from mice in (A). C) Representative western blot analysis of bulk tumor lysates (n=4 per group) from tumors in (A). **Fig 2**: Co-occurrence of TP53 mutations, DUSP4 loss, and MYC amplification in primary breast cancer. A) TCGA (Firehose legacy) analysis of the co-occurrence of TP53 mutations, DUSP4 loss, and MYC amplification, accessed from the cBioPortal Web site. B) Schema for generation of a series of cell lines derived from Dusp4FLOX PMECs carrying deletion/excision of Dusp4, deleterious p53 mutations, and MYC overexpression/amplification. **Fig** **3**: Characterization of the primary mammary epithelial cells generated from Dusp4FLOX cell line. Dusp4 functional loss of exons 3-4 (encoding the phosphatase in PMEC cells was confirmed by A) NGS and B) qRT-PCR analysis. C) Summary of all Trp53 mutations as assessed by NGS in cell lines derived from Dusp4FLOX PMECs. D) Validation of Trp53 deletion in PMEC cell lines by immunoblotting. **Fig** **4**: Low-pass WGS copy number analysis example. Log2 reads from low-pass WGS organized by genomic location. Dusp4NULL Trp53Δex7 MYCAMP demonstrate a focal amplification event (red box) including the centromeric portion of 5q, likely including the centromere. Dusp4FLOX cells are included as a visual control. **Fig 5**: Elevated mRNA expression of key genes included on the 5q amplification associated with tumorigenic potential. A) UCSC Genome browser snapshot identifying key genes included in the 5q amplification event associated with tumorigenic potential. B) mRNA expression level for key genes from (A) measured by qRT-PCR in derivative PMEC cell lines. Tu: tumorigenic potential. **Fig** **6**: Dusp4 deletion suppresses the cellular proliferation without impacting survival of PMEC cell lines. A) Time-course assessment of the proliferative capacity of Dusp4-derived PMEC cell lines by sulforhodamine B (SRB) assay. B) Dose–response assessment of survival in Dusp4-derived PMEC cell line treated with Palbociclib by SRB assay**.**

## Data Availability

Any non-commercially available reagents or data from the studies are available upon reasonable request.
