# Supplementary material for: Combined Dusp4 and p53 loss with Dbf4 amplification drives tumorigenesis via cell cycle restriction and replication stress escape in breast cancer
Source: Breast Cancer Res. 2022 Jul 18;24:51. doi: 10.1186/s13058-022-01542-y (PMC9290202; doi:10.1186/s13058-022-01542-y)

Supplementary Figure 1

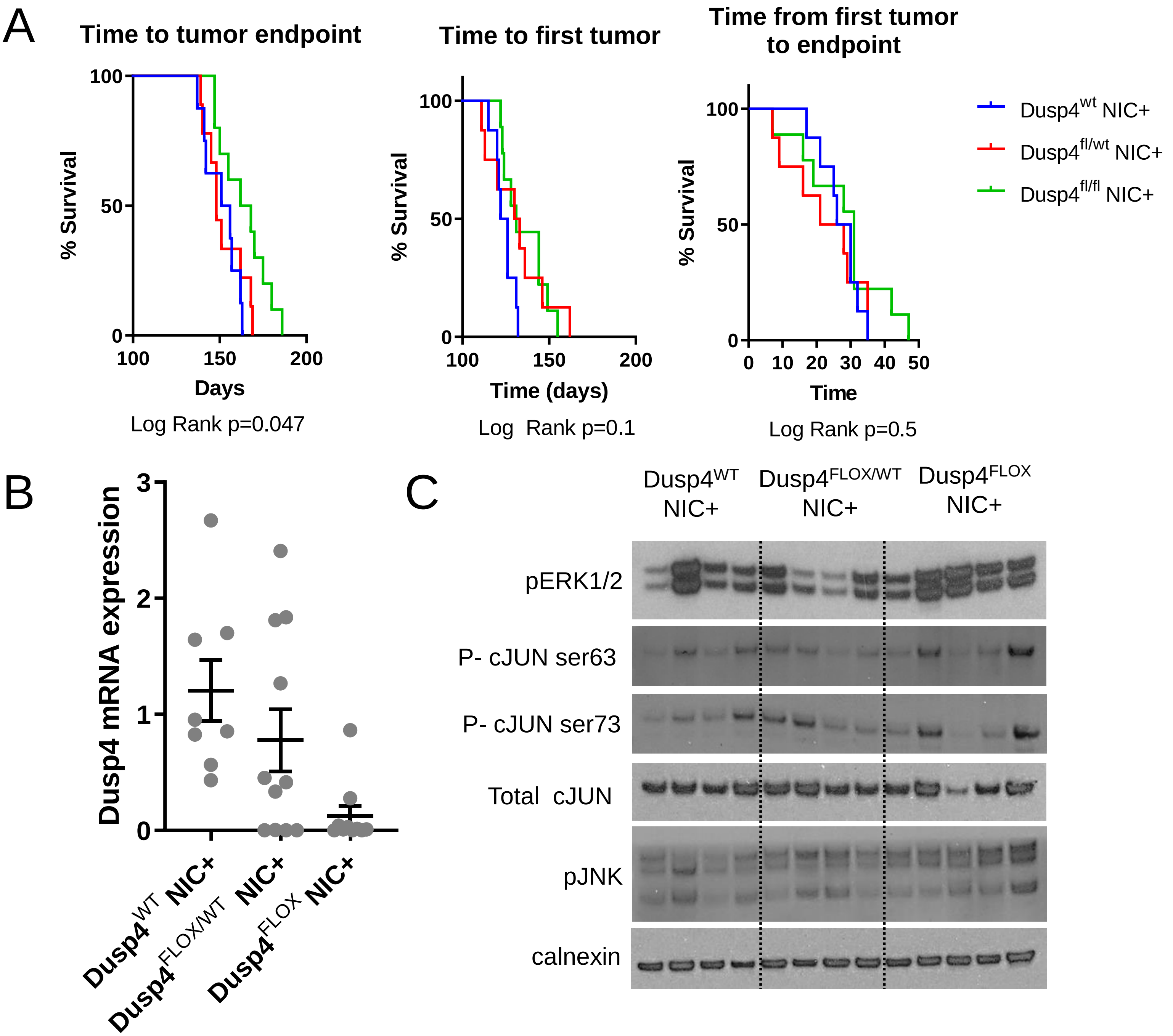

Supplementary Figure 2

A

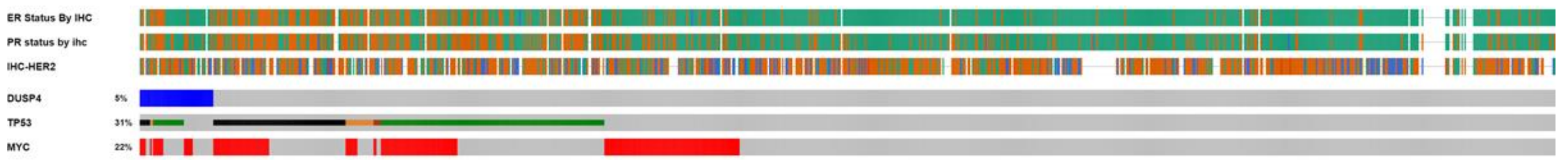

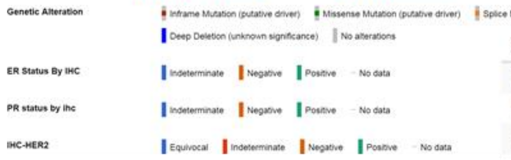

A	B	Log2 Odds Ratio	p-Value	q-Value ▲	Tendency
TP53: MUT_DRIVER	MYC: AMP	1.821	<0.001	<0.001	Co-occurrence
TP53: MUT_DRIVER	DUSP4: HOMDEL	1.867	<0.001	<0.001	Co-occurrence
DUSP4: HOMDEL	MYC: AMP	1.079	0.013	0.013	Co-occurrence

B

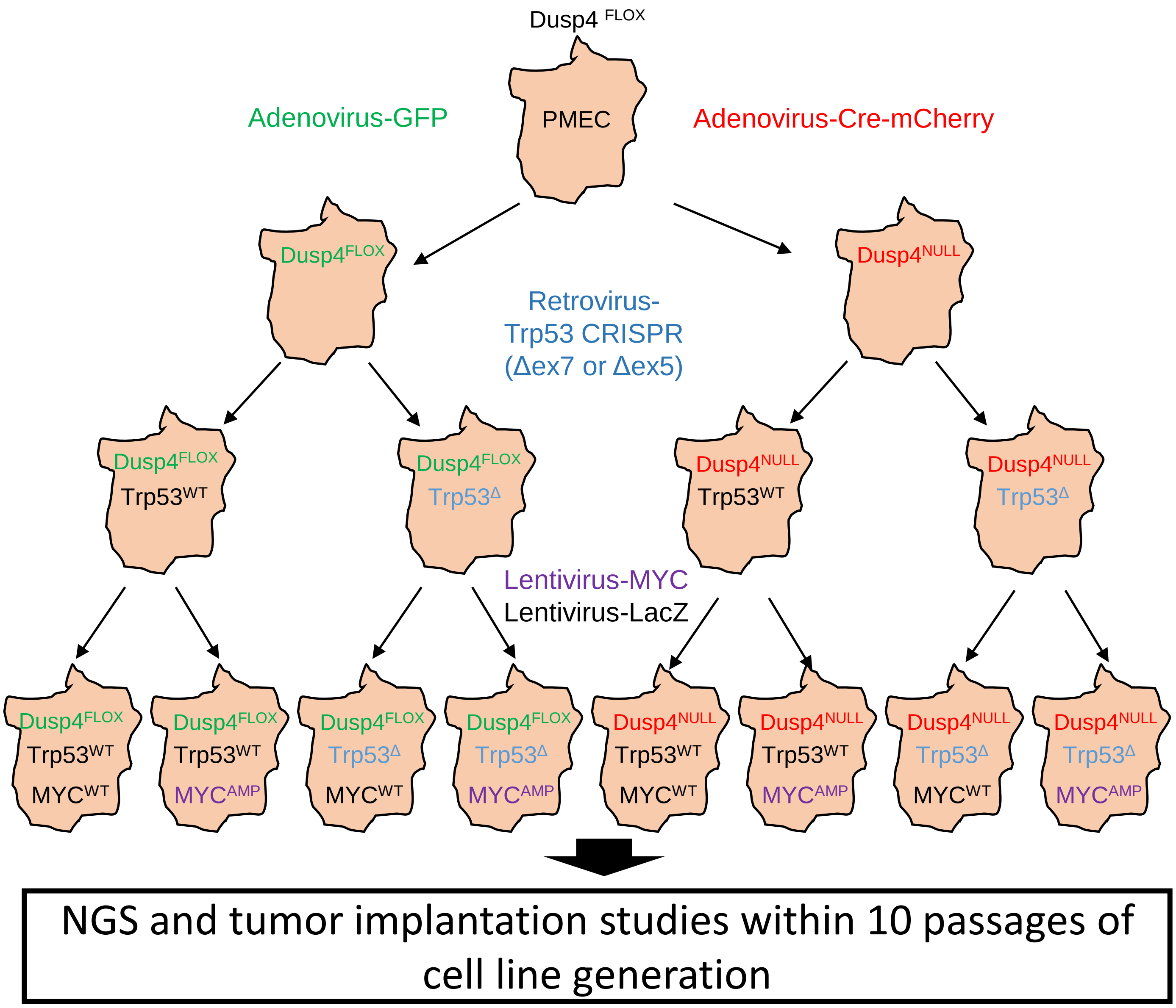

Supplementary Figure 3

A B

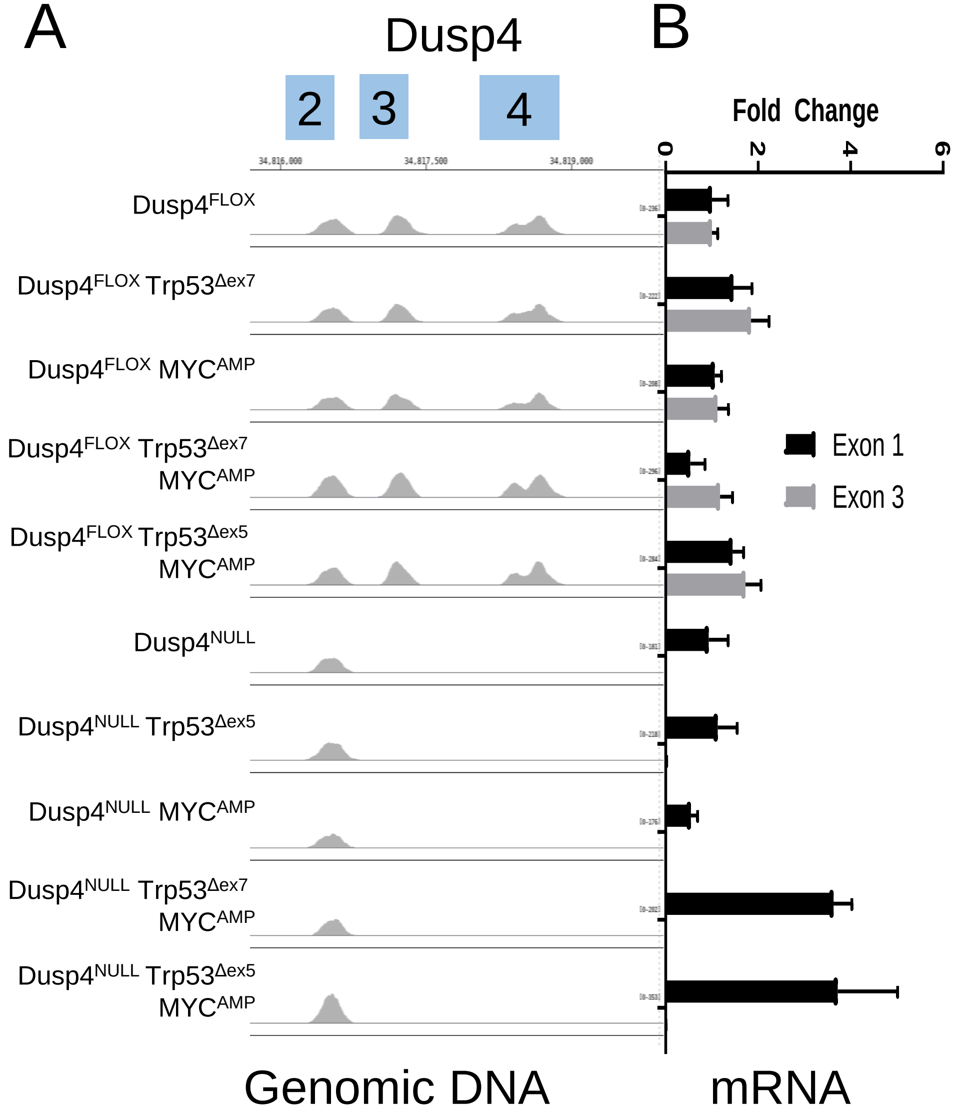

C

**Dusp4^{FLOX}**	No alterations detected
**Dusp4^{FLOX} p53$^{\Delta ex7}$**	Heterozygous (53%) stop gain (C239X) in exon 7
**Dusp4^{FLOX} MYC^{amp}**	No alterations detected
**Dusp4^{FLOX} p53$^{\Delta ex7}$ MYC^{amp}**	Homozygous (100%) frameshift insertion (C239fs) in exon 7
**Dusp4^{FLOX} p53$^{\Delta ex5}$ MYC^{amp}**	Hemizygous (23%) frameshift deletion (K162fs) in exon 5; Near-homozygous (93%) downstream non-frameshift deletion (166_167del) in exon 5
**Dusp4^{NULL}**	No alterations detected
**Dusp4^{NULL} p53$^{\Delta ex5}$**	Homozygous (100%) nonframeshift deletion (166_166del) in exon5
**Dusp4^{NULL} MYC^{amp}**	No alterations detected
**Dusp4^{NULL} p53$^{\Delta ex7}$ MYC^{amp}**	Heterozygous (45%) non-frameshift deletion (233_244del) in exon 7; Near-homozygous (83%) downstream non-frameshift deletion (237_245del) in exon 7
**Dusp4^{NULL} p53$^{\Delta ex5}$ MYC^{amp}**	Heterozygous (45%) frameshift deletion (K162fs) in exon 5; homozygous (100%) downstream non-frameshift deletion (166_167del) in exon 5

D

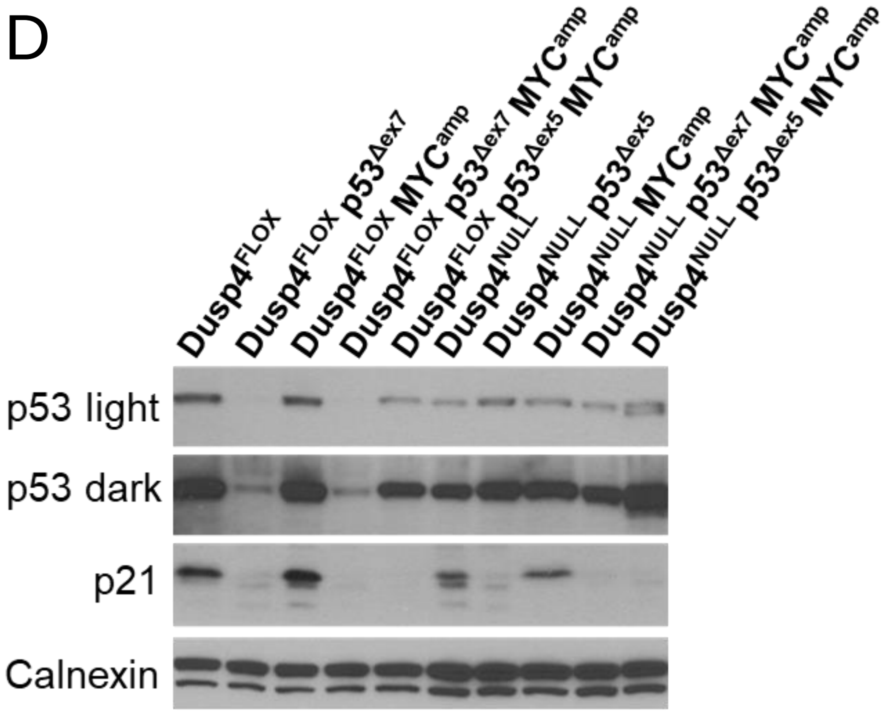

Supplementary Figure 4

A

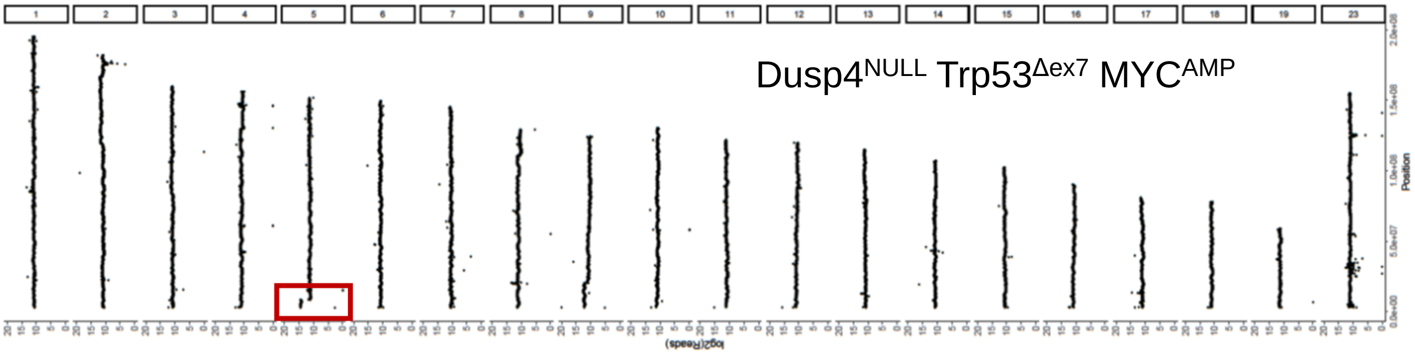

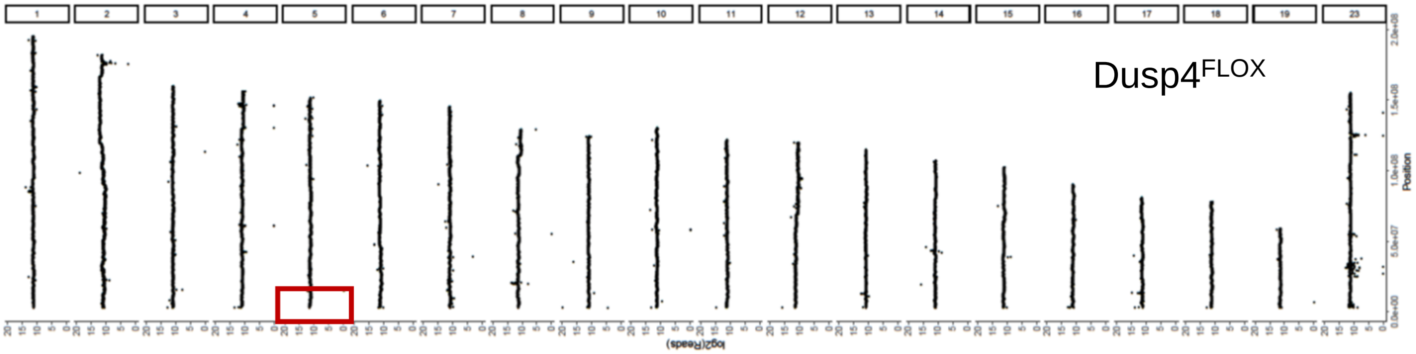

Supplementary Figure 5

A

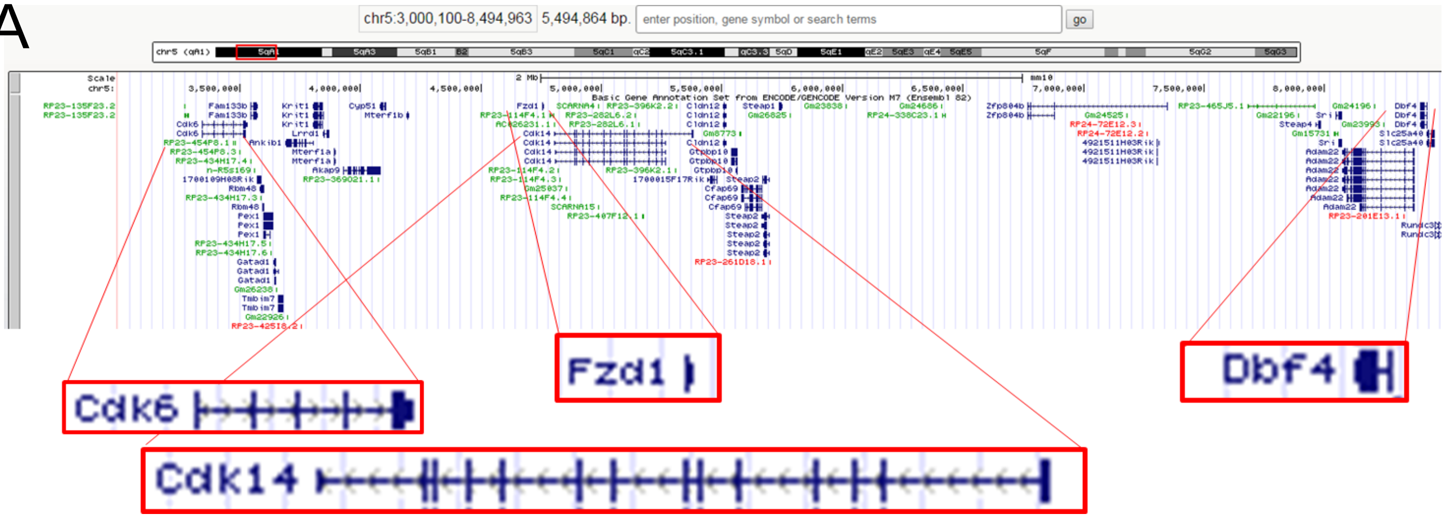

B

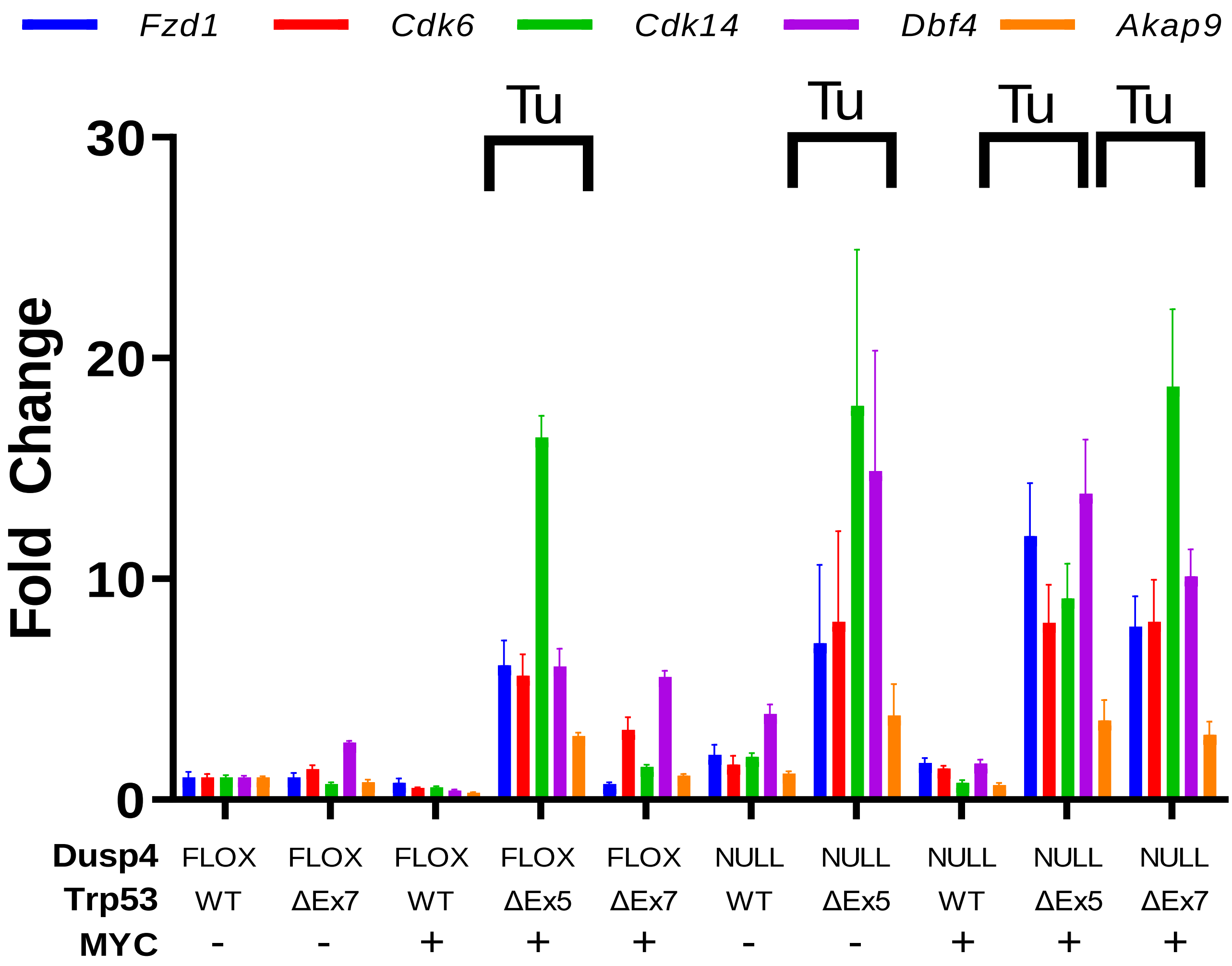

Supplementary Figure 6

A

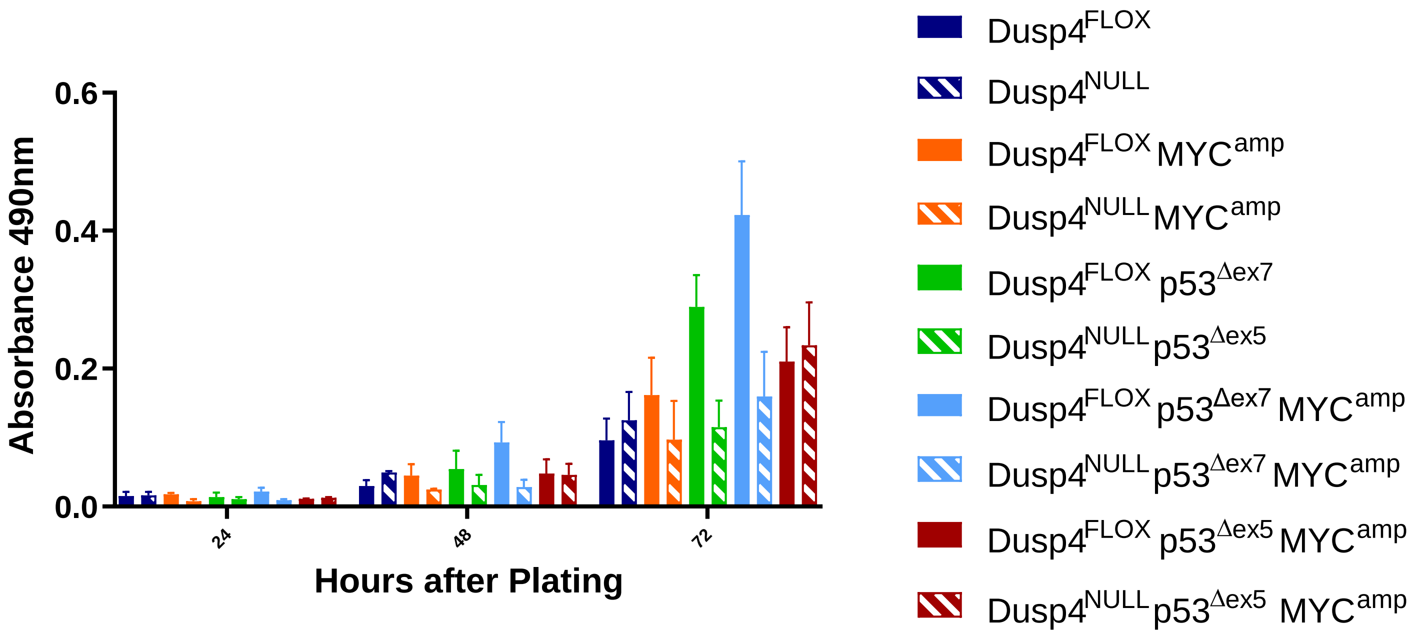

B

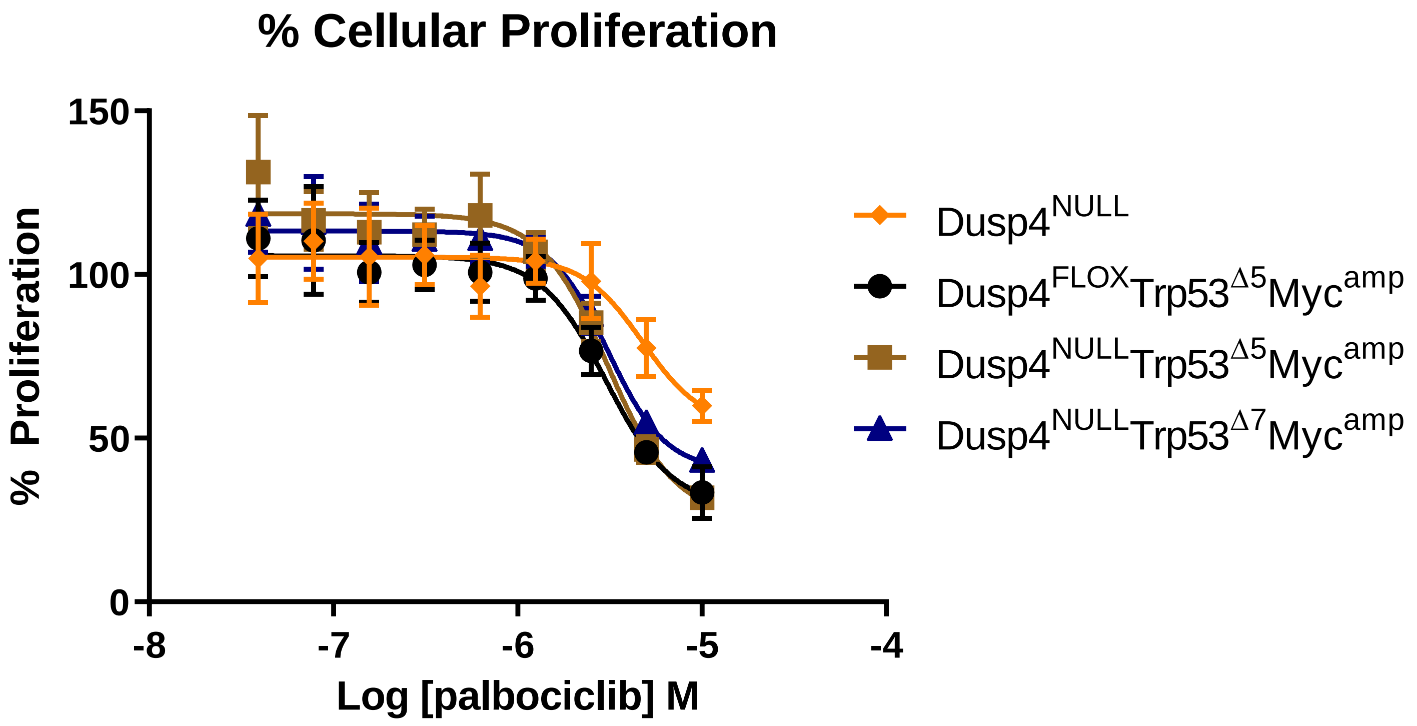

Supplement: Supplementary file 1 — Additional file 1. Fig 1: Dusp4 hemi- or homozygous deletion does not accelerate MMTV-neu-mediated tumorigenesis or growth. A) Kaplan–Meier survival of FVB/n NIC mice with Dusp4WT (n=8), Dusp4FLOX/WT (n=11), or Dusp4FLOX (n=10) alleles. Time from birth to tumor endpoint (2cm3 total tumor burden), time from birth to first palpable tumor, and time from palpable tumor to tumor endpoint are shown. B) Dusp4 mRNA expression measured from bulk tumor RNA from mice in (A). C) Representative western blot analysis of bulk tumor lysates (n=4 per group) from tumors in (A). Fig 2: Co-occurrence of TP53 mutations, DUSP4 loss, and MYC amplification in primary breast cancer. A) TCGA (Firehose legacy) analysis of the co-occurrence of TP53 mutations, DUSP4 loss, and MYC amplification, accessed from the cBioPortal Web site. B) Schema for generation of a series of cell lines derived from Dusp4FLOX PMECs carrying deletion/excision of Dusp4, deleterious p53 mutations, and MYC overexpression/amplification. Fig 3: Characterization of the primary mammary epithelial cells generated from Dusp4FLOX cell line. Dusp4 functional loss of exons 3-4 (encoding the phosphatase in PMEC cells was confirmed by A) NGS and B) qRT-PCR analysis. C) Summary of all Trp53 mutations as assessed by NGS in cell lines derived from Dusp4FLOX PMECs. D) Validation of Trp53 deletion in PMEC cell lines by immunoblotting. Fig 4: Low-pass WGS copy number analysis example. Log2 reads from low-pass WGS organized by genomic location. Dusp4NULL Trp53Δex7 MYCAMP demonstrate a focal amplification event (red box) including the centromeric portion of 5q, likely including the centromere. Dusp4FLOX cells are included as a visual control. Fig 5: Elevated mRNA expression of key genes included on the 5q amplification associated with tumorigenic potential. A) UCSC Genome browser snapshot identifying key genes included in the 5q amplification event associated with tumorigenic potential. B) mRNA expression level for key genes from (A) mea [file 13058_2022_1542_MOESM1_ESM.pdf]
